# Cilostazol protects against gastric ulcers by regulating PPAR-γ, HO-1, PECAM-1, pErk-1, NF-κB, Bcl-2, and cleaved caspase-3 protein expression

**DOI:** 10.1007/s00210-024-03176-7

**Published:** 2024-06-17

**Authors:** Nagla A. El-Shitany, Eman A. EL-saidy, Mostafa E. EL-Naggar, Samia S. Sokar

**Affiliations:** 1 Department of Pharmacology & Toxicology, Faculty of Pharmacy, Tanta University, Tanta, Egypt; 2 Department of Pharmacology & Toxicology, Faculty of Pharmacy, University of Sadat City, Sadat City, Menoufia Egypt

**Keywords:** Cilostazol, PPAR-γ, PECAM-1, pErk-1, NF-κB, Gastric ulcer

## Abstract

Millions of individuals worldwide, across all age groups, suffer from the widespread health issue of gastric ulcers. In many experiments, cilostazol (Cls), a phosphodiesterase-3 inhibitor, was recently shown to have anti-ulcer activity. Notably, Cls increases the expression and transcriptional activity of PPAR-γ in vitro and in vivo. This study aimed to evaluate the protective effect of Cls against ethanol-induced gastric ulcers and clarify the possible underlying mechanisms with an emphasis on the role of PPAR-γ. Male albino rats were treated with ethanol to induce gastric ulcers, or they were pretreated with Cls, omeprazole (Omp), GW9662, or Cls + GW9662 for 14 consecutive days before receiving ethanol. Cls protects against ethanol-induced gastric ulcers. Cls treatment significantly reduced ethanol-induced upregulation of the pro-inflammatory markers (IL-1β, IL-6, TNF-α, and NF-κB), MDA (a marker of lipid peroxidation), and caspase-3 and cleaved caspase-3 (apoptotic markers). On the other hand, Cls treatment counteracted ethanol-induced downregulation of PPAR-γ, pErk-1, HO-1 and GSH (antioxidant markers), PECAM-1 and NO (healing markers), and Bcl-2 (antiapoptotic marker). However, when combined with GW9662, a potent antagonist of PPAR-γ, Cls loses its effects. In conclusion, these results suggest that PPAR-γ and pErk-1 are essential for Cls’s protective effects against ethanol-induced gastric ulcers.

**Graphical abstract:**

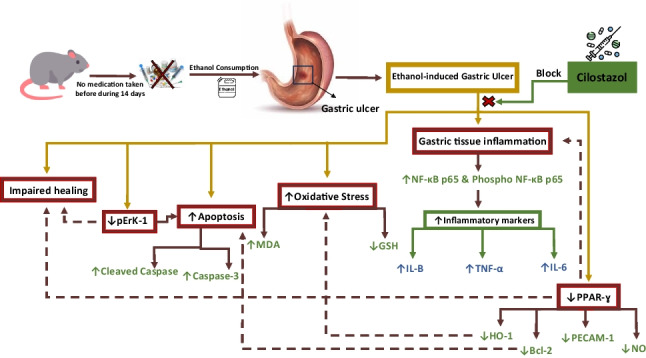

## Introduction

Gastric ulcers (GU), a type of peptic ulcer disease, is a significant public health problem worldwide, affecting about 5–10% of people at some point in their lives, making it a major public health burden in the twenty-first century (Lanas and Chan 2017). Ethanol-induced GU is a versatile animal model that can be used to investigate the mechanisms of action and therapeutic potential of new anti-ulcer drugs (Arab et al. 2015). In the current study, ethanol was used due to its high ulcerogenic effect, which is considered a major risk factor. Ethanol devastates almost every aspect of gastroprotection via various mechanisms, including gastric acid elevation, production of ROS, release of inflammatory cytokines, leukocyte infiltration, and induction of programmed cell death leading to tissue injury (Moawad et al. 2019). The proposed mechanism of ethanol-inducing gastric ulcer occurs via gastric wall penetration and digestion due to its hydrolytic and proteolytic action, as well as endothelial cell damage, as it reduces gastric blood flow (Rahman et al. 2020). Ethanol damages the gastric mucosa by reducing bicarbonate, mucus, and nitric oxide (NO) secretion, reducing blood flow, and inducing oxidative stress and inflammation (El-Maraghy et al. 2015). Moreover, ethanol-induced GU is associated with a significant decrease in the production of the cytoprotective protein peroxisome proliferator-activated receptor gamma (PPAR-γ) (Arab et al. 2022).

PPAR-γ is a nuclear receptor that regulates many gene expressions and is involved in controlling inflammation and the immune system (Auwerx 1999). In oxidative stress conditions, the nuclear receptor PPARγ directly regulates a vast array of genes involved in response to oxidative stress and exerts anti-inflammatory effects trans-repressing nuclear factor kappa B (NF-κB). These genes involve heme oxygenase-1 (HO-1) and the antiapoptotic protein B-cell lymphoma 2 (Bcl-2) (Polvani et al. 2012). PPAR-γ controls the release of inflammatory cytokines from damaged tissue and immune cells, such as tumor necrosis factor-alpha (TNF-α) and interleukin-1 beta (IL-β) (Su et al. 1999). Reduced level of the PPAR-γ protein is involved in the formation of GU (Mahmoud-Awny et al. 2015). PPAR-γ activators protect the stomach and speed up ulcer healing in many experimental models of GU (Lahiri et al. 2009; Saha 2015).

Extracellular-regulated kinase (Erk) is a serine/threonine protein kinase that is part of the MAPK signaling pathway, which consists of three main cascades: Raf-1, Erk, and p38 MAPK (Santen et al. 2002). pErk 1/2 can modulate many different proteins to control gene expression, cell proliferation, apoptosis, differentiation, cell-matrix interactions, and cell migration (Mandal et al. 2014). Studies have shown that blocking pErk slows down stomach ulcer healing. Interestingly, when the body is under oxidative stress, increasing pErk activity might actually help by reducing apoptotic cell death signals or preventing another Akt activation (Hu et al. 2019; Luo et al. 2016; Wang et al. 2020).

Cilostazol (Cls), a specific phosphodiesterase type-3 inhibitor, inhibits the degradation of cAMP, increasing its intracellular level, and subsequently activates protein kinase A. It is clinically used in the treatment of peripheral vascular disorders (Choi et al. 2018; Sakamoto et al. 2018). The therapeutic efficacy of Cls has been attributed to its vasodilatory effects in NO-dependent and NO-independent pathways (Chi et al. 2008), as well as its inhibitory effect on the release of pro-inflammatory cytokines such as IL-1β, IL-6, and TNF-α (Asal and Wojciak 2017). Moreover, Cls demonstrated partial agonist features on the cytoprotective signal PPAR-γ. Cls increases PPAR-γ expression and activity in various cells, including those in the kidneys, retinas, and hearts of diabetic and ischemic animals (Wang et al. 2008; Park et al. 2009; Biscetti et al. 2013; Ragab et al. 2014).

Recent studies have shown that Cls protects against ulcers in many animal models, including ethanol-induced ulcers (Rashad et al. 2019; Moawad et al. 2019; Imad and Al-Qadh 2022). However, no studies have yet investigated the role of PPAR-γ and pErk-1 in Cls’s gastroprotective effects. This study aimed to investigate the protective effect of Cls against ethanol-induced GU and to focus on a better understanding of the mechanistic pathways through which it may elicit its impact, emphasizing the role of PPAR-γ, pErk-1, and apoptosis.

## Materials and methods

### Drugs and chemicals

Cls was purchased as 100 mg Cls tablets, Otsuka Pharmaceutical Co. SAE, Egypt; omeprazole (Omp) was purchased as 40 mg Omp capsules, Hikma Pharmaceuticals Industries SAE, Egypt; and GW9662 was purchased from Thermo Fisher (Kandel) GmbH Erlenbachweg 276870 Kandel, Germany. Cls, Omp, and Gw9662 were prepared in dimethyl sulfoxide (DMSO).

### Experimental animals

In this study, 30 adult male albino rats weighing 180–200 g were used. The rats were kept at a temperature of 24 ± 2 °C and a humidity of 60 ± 10% during a 12:12 h dark/light cycle. Before the induction of ulcers, all rats were allowed free access to tap water during a 36-h fast. To prevent coprophagy, rats were housed in large cages with wire bottoms. The Research Ethics Committee of the Faculty of Pharmacy, Tanta University, Tanta, Egypt, approved this study (TP/RE/5/23 M-0019). The research was carried out according to the National Research Council’s Guide for the Care and Use of Laboratory Animals.

#### Experimental design

**Fig. 1:**
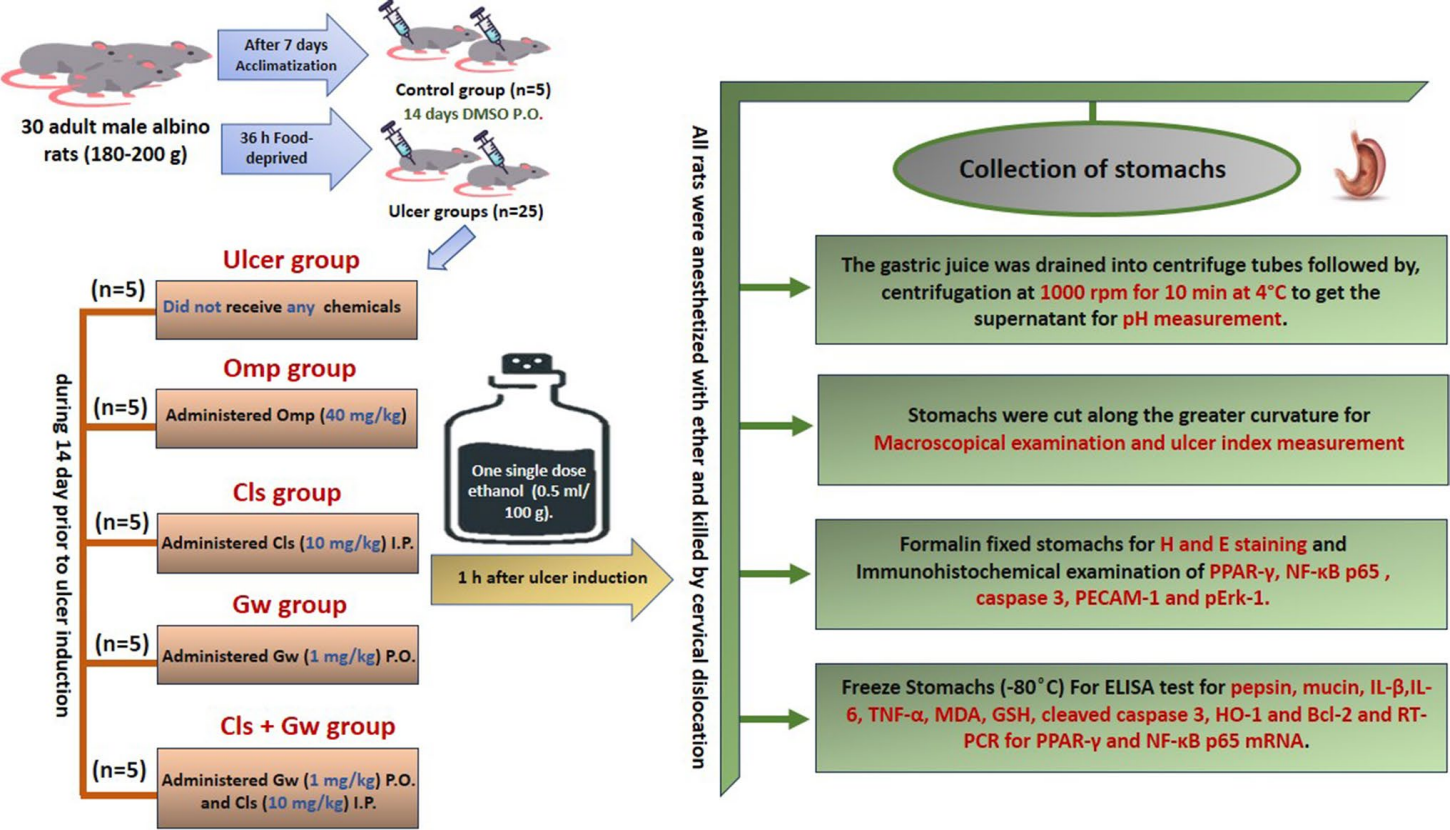
Experimental design and sample collection

Figure 1 illustrates the experimental design and sample collection. Rats were randomly assigned to six groups (*n* = 5). (1) Control group: rats in this group were administered DMSO P.O., (2) ulcer group: rats in this group were administered a single dose of ethanol (0.5 ml/100 g body weights) P.O. (Wu et al. 2018), (3) Omp group: rats in this group were administered Omp (40 mg/kg) P.O. (Hu et al. 2020), (4) Cls group: rats in this group were administered Cls (10 mg/kg) I.P. (Hu et al. 2020), (5) Gw group: rats in this group were administered GW9662 (1 mg/kg) P.O. (Elshazly et al. 2018), and (6) Cls + GW group: rats in this group were administered Gw9662 (1 mg /kg) P.O. and Cls (10 mg/kg) i.p. All treatments were given for 14 days before ulcer induction with ethanol (Ragab et al. 2014). The ulcer was induced in all the experimental groups except the control group. Rats in the ulcer group did not receive any vehicles before ethanol. All rats were fasted for 36 h before ulcer induction (Chen et al. 2005).

### Sample collection

An hour after ulcer induction, rats were anesthetized with ether, killed by cervical dislocation and the stomachs were excised immediately. Subsequently, gastric juice was collected for the pH measurement. Next, the stomachs were opened, rinsed with 0.9% saline, and subjected to gross macroscopic examination for ulcer scoring and index calculation. Stomachs were gathered on liquid nitrogen for the biochemical analysis and stored at −80 °C. For the histopathologic and immunohistochemistry examination, stomachs were fixed in 10% neutral buffered formalin.

### Macroscopic examination

The freshly excised stomachs were examined macroscopically for hemorrhagic lesions in the glandular mucosa. Soon after the animals were sacrificed, their stomach were dissected out and cut along the greater curvature, and the mucosa were rinsed with cold normal saline to remove blood contaminants. The length (mm) of each lesion was measured (Bozkurt et al. 1998). The severity of mucosal lesions was scored as ulcer index according to the following scale: 0 = no pathology; 1 = a small ulcer (1–2 mm); 2 = a medium ulcer (3–4 mm); 4 = a large ulcer (5–6 mm); and 8 = a larger ulcer (˃6 mm). The sum of the total scores divided by the number of rats was expressed as the mean ulcer index (UI) (Das and Banerjee 1993).

### Determination of the gastric pH

The gastric content of the stomachs was drained into centrifuge tubes and subsequently centrifuged for 10 min at 1000 rpm for 10 min at 4 °C to get the supernatant, and the supernatant was assessed for pH measurement (Sivaraman and Muralidharan 2011; Gupta et al. 2021). Then, an aliquot of 1 ml gastric juice was diluted with 1 ml of distilled water, and the pH of the solution was measured using a pH meter (HANNA® bench top pH meters, HI 110, USA) (Dashputre and Naikwade 2011).

### Histopathological examination

The formalin-fixed stomachs were dehydrated with different concentrations of ethanol. Stomachs were then embedded in paraffin film and cut into 5-μm sections. Subsequently, the stomachs were stained with hematoxylin and eosin (H&E) and the slides were used for the histopathological microscopic examination (Burchette 2009).

### Preparation of stomach homogenates

Stomachs were homogenized in 0.05 M phosphate buffer (pH 7) to prepare 10% of the homogenate using a polytron homogenizer (PT-MR 3100, kinematica AG, Littau, Switzerland) at 4 °C. After centrifuging the stomach homogenates for 20 min at 10,000 rpm, the supernatant was stored at −80 °C.

### Quantification of stomach protein contents

Using a protein estimate kit from Genei, Bangalore, India, the stomachs’ protein content was measured using the Bradford technique (Bradford 1976).

### Quantification of stomach pepsin and mucin contents

Stomach pepsin and mucin contents were measured using the rat pepsin ELISA kit (Catalog No: NBP2-82494), Novus Biologicals, USA, and the rat mucin-2, MUC2 ELISA kit (Catalog No: BT-E1770Ra), ImuGeX GmbH, Germany, respectively, as directed by the kit’s instructions.

### Quantification of stomach oxidative stress markers and nitric oxide

Stomach malondialdehyde (MDA) contents were measured using the MDA colorimetric/fluorometric assay kit (Catalog No: K739), BioVision, Inc, USA. Reduced glutathione (GSH) contents were measured using the GSH colorimetric kit (Catalog No: K464), BioVision, Inc, USA. HO-1 contents were measured using the rat HO-1 ELISA kit (Catalog No: E0676Ra), BT LAB, China. NO contents were measured using the NO colorimetric kit (Catalog No: K262), BioVision, Inc, USA. All markers were measured as directed by the kit’s instructions.

### Quantification of stomach inflammatory cytokines

TNF-α, IL-1β, and IL-6 contents were measured using the rat TNF-α BioLegend’s ELISA MAX™ Deluxe Set (Catalog No: 438204), BioLegend, Inc, CA, the rat IL-1β ELISA kit (Catalog No: SEA563Ra), Cloud-Clone Corp, USA, and the rat IL-6 ELISA kit (Catalog No: SEA079Ra), Cloud-Clone Corp, USA, respectively, as directed by the kit’s instructions.

### Real-time polymerase chain reaction (RT-PCR) investigation

RT-PCR was adopted to investigate stomach PPAR-γ and NF-κB p65 mRNA expressions. Briefly, Direct-zol RNA Miniprep Plus (Catalog No. R2072, Zymo Research Corp, USA) was used to prepare the stomach homogenates in order to acquire total RNA. The acquired RNA was reverse-transcribed using the SuperScriptTM IV One-Step RT-PCR System (Catalog No. 12594100, Thermo Fisher Scientific, USA), and then PCR was conducted in a single phase. The reaction mix samples that were produced later used as templates for RT-PCR (Step One Applied Biosystem, Foster, USA). By normalizing each target gene’s expression to the GADPH level, the relative quantification of its expression was carried out. The 2-ΔΔCT method was used to accomplish the relative quantification. Table 1 contains a list of primer sequences utilized in this investigation.

**Table 1:** The list of the RT-PCR technique’s primer sequences

	Forward sequence	Reverse sequence	Gene accession number
*PPARγ*	CGAGTGCCGAGTCTGTGGGGATA	ATGGTGATTTGTCTGTTGTCTTTC	AB019561.1
*NFκB p65*	GTCTCAAACCAAACAGCCTCAC	CAGTGTCTTCCTCGACATGGAT	NM_199267.2
*GAPDH*	GGCAAGGTCATCCCAGAGCT	CCCAGGATGCCCTTTAGTGG	DQ40057.1

### Quantification of stomach apoptotic and antiapoptotic markers

Cleaved caspase-3 contents were measured using the rat cleaved caspase-3 ELISA kit (Catalog No: SL1366Ra), SunLong Biotech Co., LTD, China. Bcl-2 contents were measured using the rat Bcl-2 ELISA kit (Catalog No: SEA778Ra), Cloud-Clone Corp, USA. Caspase-3 was measured using the immunohistochemical investigation.

### Immunohistochemical investigation

The immunohistochemical procedure was adopted to investigate stomach, NF-κB p65, PPAR-γ, caspase-3, pErk-1, and PECAM-1 protein expression using the technique described by Khalil et al. (2020). For the purpose of retrieving antigens, sections were soaked in a 0.05 M citrate buffer solution pH 6.8 after being waxed. After that, these areas were exposed to protein block and 0.3% H2O2. Sections were then incubated with anti-NF-κB p65 monoclonal antibody (Catalog No: (F-6): sc-8008, Santa Cruz Biotechnology, USA, 1:100 dilution), anti-PPAR-γ monoclonal antibody (Catalog No: (E-8): sc-7273, Santa Cruz Biotechnology, USA, 1:200 dilution), anti-caspase-3 polyclonal antibody (Catalog No: PA5-77887, Invitrogen, USA, 1:100 dilution), anti-pErk-1 polyclonal rabbit antibody (Catalog No: PA5-40294, Invitrogen, USA, 1:20 dilution), and anti-PECAM-1 monoclonal antibody (Catalog No: (H-3): sc-376,764, Santa Cruz Biotechnology, USA, 1:200 dilution). After rinsing the slides with phosphate-buffered saline, they were treated for 30 min at room temperature with a goat anti-rabbit secondary antibody (Catalog No. K4003, EnVision+TM System Horseradish Peroxidase Labelled Polymer, Dako, USA). The DAB kit (Catalog No. ab64238, Abcam, UK) was used to view the slides, and Mayer’s hematoxylin was used as a counterstain.

### Statistical study

The findings were statistically analyzed using GraphPad Prism version 8. The one-way ANOVA test and the Tukey-Kramer post hoc test were used to compare the final results between the study groups. A threshold of significance of *p* < 0.05 was established. The data was displayed as mean ± SD.

## Results

### Effect of Cls and Cls + Gw on the macroscopic appearance of gastric mucosa, gastric UI, and gastric pH

**Fig. 2:**
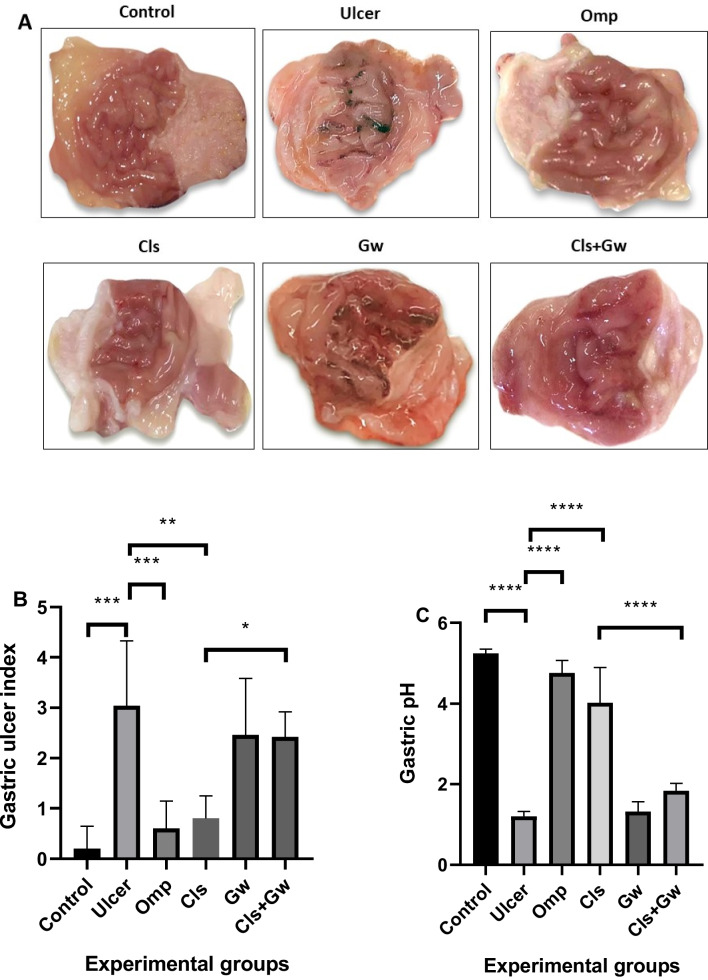
Effect of Cls and Cls + Gw on the macroscopic appearance of gastric mucosa (**A**), gastric ulcer index (**B**), and gastric pH (**C**). **A** Representative camera images of the gastric mucosa. For **B** and **C **Results were presented as mean ± SD (*n* = 5 rats/group). ^*^Significance confirmed at *p* < 0.05. ^**^Significance confirmed at *p* < 0.01. ^***^Significance confirmed at *p* < 0.001. ^****^Significance confirmed at *p *< 0.0001. Cls, cilostazol; Omp, omeprazole; Gw, GW9662

Figure 2A illustrates the macroscopic appearance of gastric mucosa for each group. The ethanol (ulcer) group had notable glandular mucosal hemorrhagic lesions (HL) compared to the normal control group. When compared to the ulcer group, Omp and Cls significantly protected the glandular mucosa against ethanol-induced HL. In contrast, the coadministration of Gw with Cls markedly increased glandular mucosa HL compared to the Cls group. Figure 2B illustrates the gastric UI for each research group. The ethanol (ulcer) group had a significantly higher UI than the control group. When compared to the ulcer group, Omp and Cls significantly decreased UI. In contrast, the coadministration of Gw with Cls significantly increased UI compared to the Cls group. Figure 2C illustrates the gastric pH for each research group. The ethanol (ulcer) group had a significantly lower gastric pH than the control group. When compared to the ulcer group, Omp and Cls significantly increased pH. In contrast, the coadministration of Gw with Cls significantly decreased pH compared to the Cls group.

### Effect of Cls and Cls + Gw on gastric pepsin and mucin contents

**Fig. 3:**
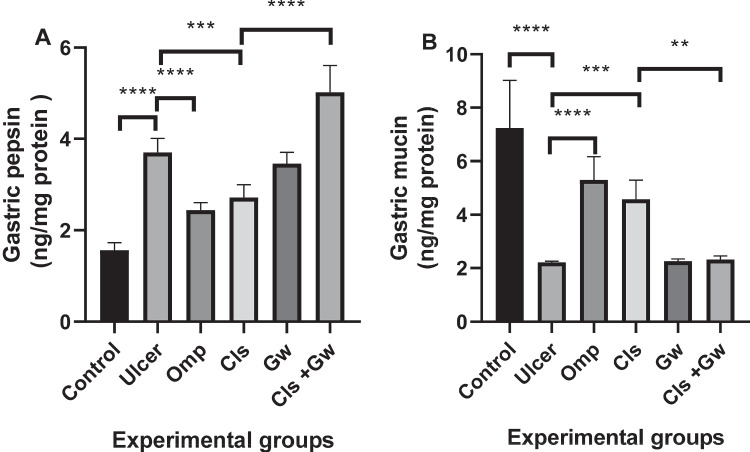
Effect of Cls and Cls + Gw on gastric pepsin and mucin contents (**A** pepsin and **B** mucin). Results were presented as mean ± SD (*n* = 5 rats/group). ^**^Significance confirmed at *p *< 0.01. ^***^Significance confirmed at *p *< 0.001. ^****^Significance confirmed at *p *< 0.0001. Cls, cilostazol; Omp, omeprazole; Gw, GW9662

Figure 3A and B illustrate the gastric pepsin and mucin contents for each research group, respectively. The ethanol (ulcer) group demonstrated a significant rise in pepsin concentrations as well as a significant reduction in the gastric mucin contents compared to the control group. The observed alterations in pepsin and mucin were significantly improved in the Omp and Cls groups compared to the ulcer group. On the other hand, coadministration of Gw with Cls significantly boosted pepsin concentrations while decreasing mucin contents compared to the Cls group.

### Effect of Cls and Cls + Gw on histopathology of the gastric mucosa

**Fig. 4:**
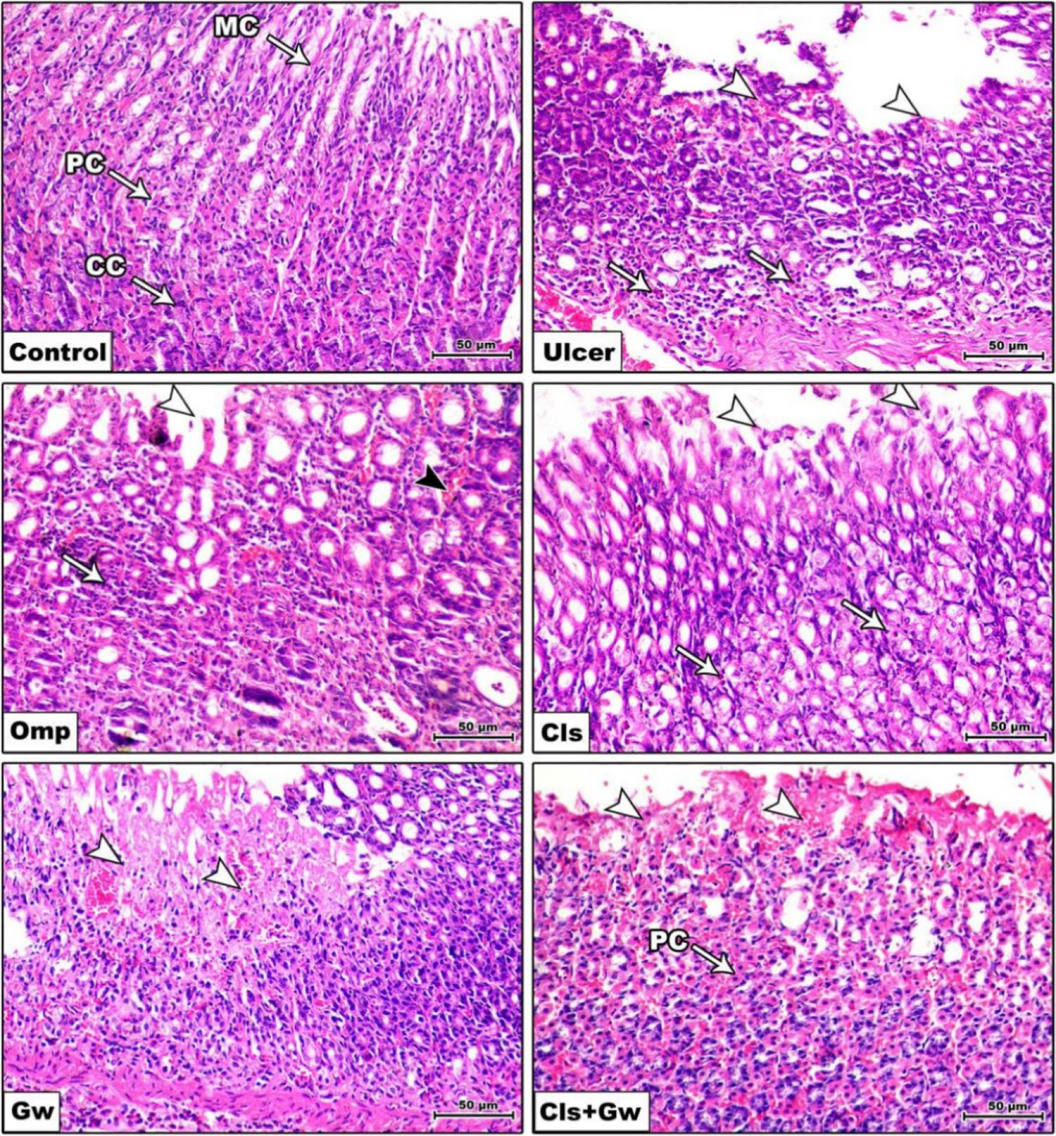
Effect of Cls and Cls + Gw on histopathology of the gastric mucosa (representative images of gastric mucosa for each group, H&E ×200, bar = 50 μm). Cls, cilostazol; Omp, omeprazole; Gw, GW9662 (MC mucosal cells, PC parietal cells, and CC chief cells)

Figure 4 illustrates the gastric mucosa histopathological alterations for each research group. The photo of the control group showed normal gastric mucosa consisting of normal gastric glands with normal mucous cells, parietal cells, and chief cells. The ethanol (ulcer) group photo showed mucosal ulceration associated with hemorrhage within the mucosa and features of gastritis associated with inflammatory cell infiltration, mostly mononuclear cells with plenty of eosinophils. The photo of the Cls group showed decreased mucosal ulceration associated with mild eosinophilic inflammatory cell infiltration. Similarly, the photo of the Omp group showed decreased mucosal ulceration associated with decreased congestion of the blood vessels, necrosis, and inflammatory cell infiltration. In contrast, the Gw pretreated Cls group photo showed degenerative and necrotic changes in the apical portion of the gastric mucosa and normal parietal cells.

### Effect of Cls and Cls + Gw on gastric tissues oxidative stress markers (MDA, GSH, and HO-1)

**Fig. 5:**
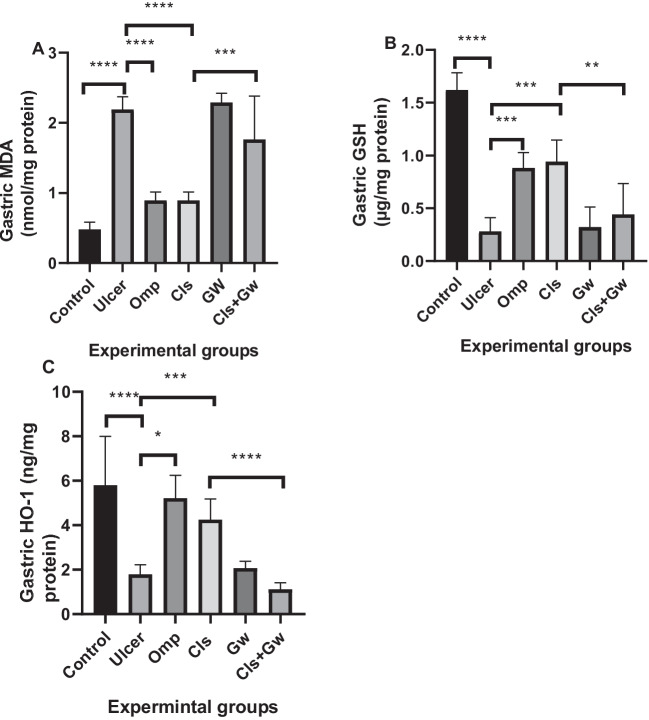
Effect of Cls and Cls + Gw on gastric tissues oxidative stress markers (**A** malonaldehyde (MDA), **B** reduced glutathione (GSH), and **C** heme oxygenase-1 (HO-1)). Results were presented as mean ± SD (*n* = 5 rats/group). ^*^Significance confirmed at *p *< 0.05. ^**^Significance confirmed at *p *< 0.01. ^***^Significance confirmed at *p *< 0.001. ^****^Significance confirmed at *p *< 0.0001. Cls, cilostazol; Omp, omeprazole; Gw, GW9662

Figure 5 illustrates the gastric tissue contents of the oxidative stress markers (MDA Fig. 5A, GSH Fig. 5B, HO-1 Fig. 5C) for each research group. Compared to the control group, the ethanol (ulcer) group revealed a significant rise in MDA content in stomach tissue and a significant drop in both GSH and HO-1 contents. Compared to the ulcer group, the Cls and Omp groups had a significant reduction in MDA content and a significant rise in both GSH and HO-1 contents. Conversely, the Gw pretreated Cls group showed a significant increase in MDA contents and significant decreases in GSH and HO-1 contents compared to the Cls group.

### Effect of Cls and Cls + Gw on gastric tissues inflammatory markers (IL-1β, IL-6, and TNF-α)

**Fig. 6:**
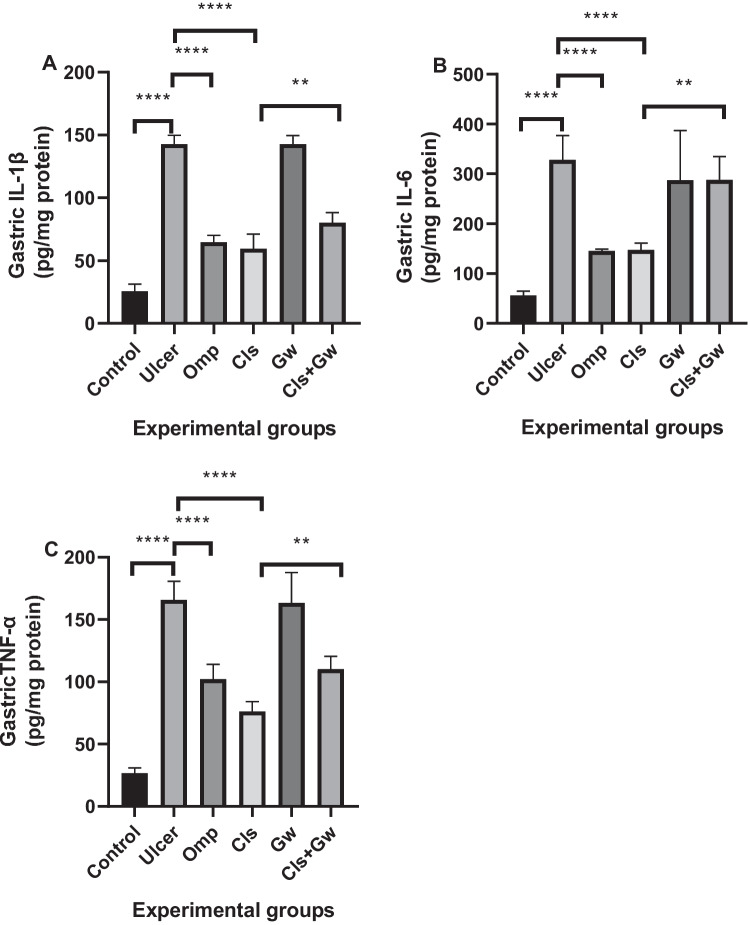
Effect of Cls and Cls + Gw on gastric tissues inflammatory markers (**A** interleukin-1beta (IL-1β), **B** interleukin-6 (IL-6), and **C** tumor necrosis factor-alpha (TNF-α)). Results were presented as mean ± SD (*n* = 5 rats/group). ^**^Significance confirmed at *p* < 0.01. ^****^Significance confirmed at *p *< 0.0001. Cls, cilostazol; Omp, omeprazole; Gw, GW9662

Figure 6 illustrates the gastric tissue contents of the inflammatory markers (IL-1β Fig. 6A, IL-6 Fig. 6B, and TNF-α Fig. 6C) for each research group. Compared to the control group, the ethanol (ulcer) group revealed significant rises in IL-1β, IL*-*6, and TNF-α contents in stomach tissue. Compared to the ulcer group, the Cls and Omp groups had significant reductions in IL*-*1β, IL*-*6, and TNF-α contents. Conversely, the Gw pretreated Cls group showed significant increases in IL-1β, IL-6, and TNF-α contents compared to the Cls group.

### Effect of Cls and Cls + Gw on gastric tissues NF-κB p65 mRNA and protein expression of phospho-NF-κB p65

**Fig. 7:**
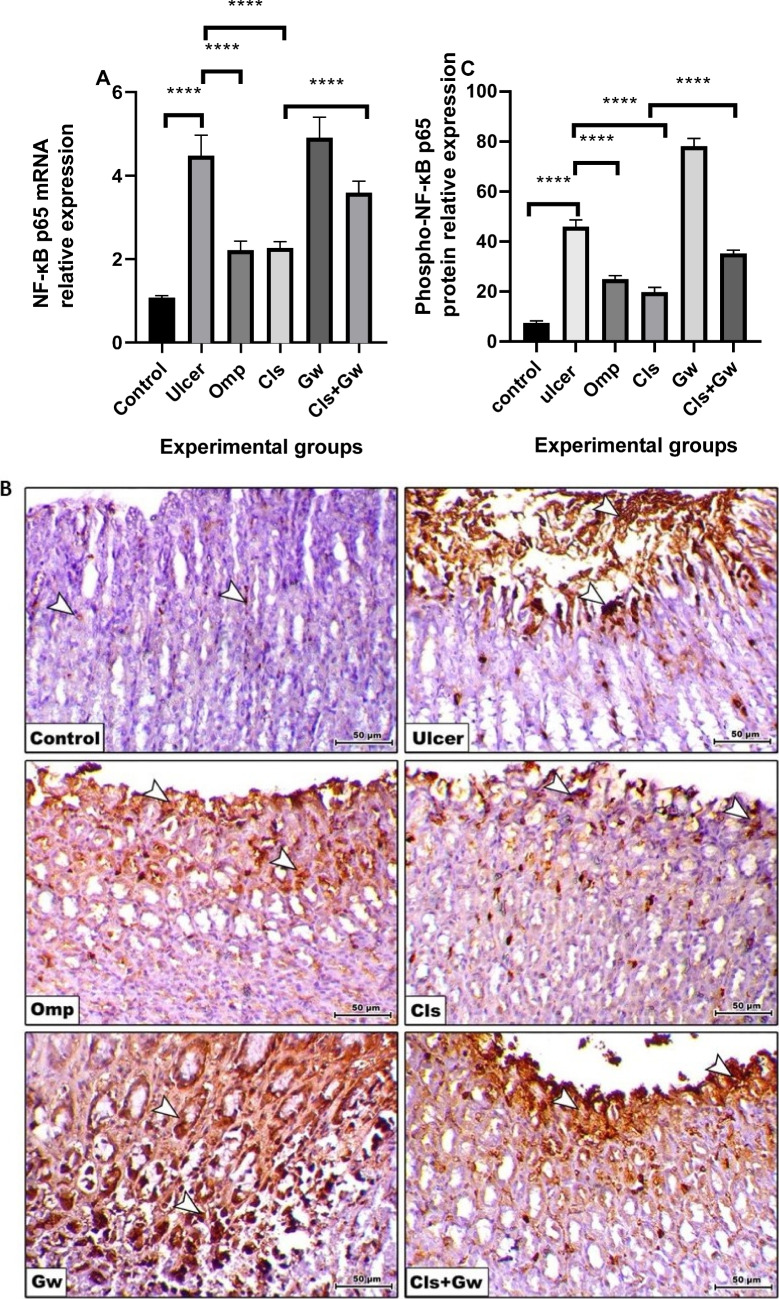
Effect of Cls and Cls + Gw on gastric tissues NF-κB p65 mRNA and phospho-NF-κB p65 protein expression. **A** RT-PCR quantification of NF-κB p65 mRNA; **B** immunohistochemical quantification of phospho-NF-κB p65 protein (×200, bar = 50 μm); **C** Image J quantification of phospho-NF-κB p65 protein. Results were presented as mean ± SD (*n* = 5 rats/group). ^****^Significance confirmed at *p* < 0.0001. Cls, cilostazol; Omp, omeprazole; Gw, GW9662

Figure 7A illustrates the gastric tissue NF-κB p65 mRNA while Fig. 7B and C illustrate protein expression of phospho-NF-κB p65 for each research group. Compared to the control group, the ethanol (ulcer) group revealed significant rises in gastric NF-κB p65 mRNA and phospho-NF-κB p65 protein expression. Compared to the ulcer group, the Cls and Omp groups produced significant reductions in gastric NF-κB p65 mRNA and phospho-NF-κB p65 protein expression. Conversely, the Gw pretreated Cls group showed a significant increase in gastric NF-κB mRNA and phospho-NF-κB p65 protein expression compared to the Cls group.

### Effect of Cls and Cls + Gw on gastric tissues PECAM-1 protein expression and NO contents

**Fig. 8:**
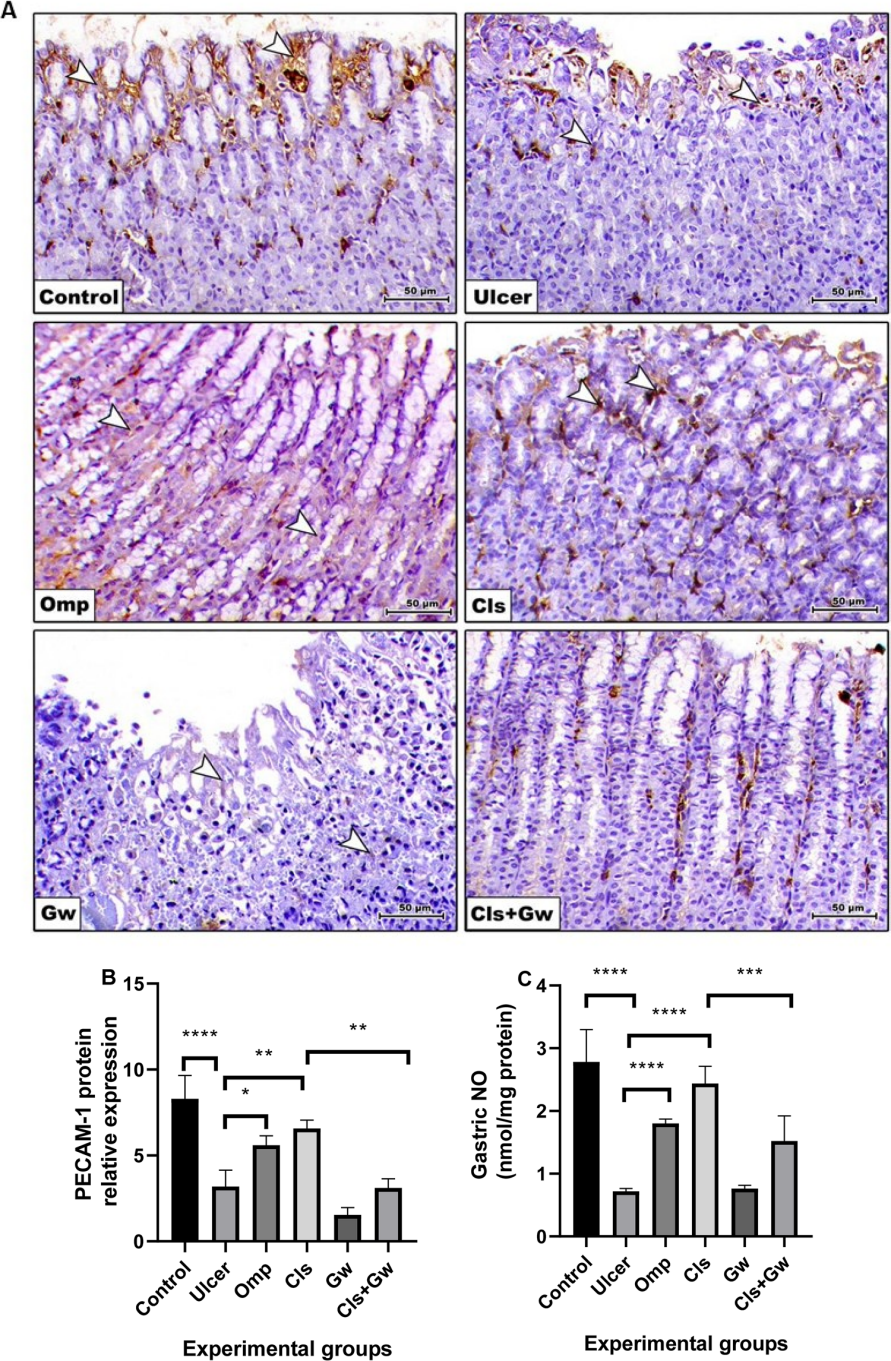
Effect of Cls and Cls + Gw on gastric tissues PECAM-1 protein expression (**A** and **B**) and nitric oxide content (**C**). **A** Immunohistochemical quantification of PECAM-1 (×200, bar = 50 μm). **B** Relative expression of PECAM-1 (Image J quantification). Results were presented as mean ± SD (*n* = 5 rats/group). ^*^Significance confirmed at *p* ˂ 0.05. ^**^Significance confirmed at *p* < 0.01. ^***^Significance confirmed at *p* < 0.001. ^****^Significance confirmed at *p* < 0.0001. Cls, cilostazol; Omp, omeprazole; Gw, GW9662

Figure 8A and B illustrate the gastric tissue PECAM-1 immunoexpressions for each research group. Compared to the control group, the ethanol (ulcer) group revealed a significant decrease in gastric PECAM-1 immunoexpression. Compared to the ulcer group, the Cls and Omp groups produced significant increases in gastric PECAM-1 immunoexpression. Conversely, the Gw pretreated Cls group showed a significant decrease in gastric PECAM-1 immunoexpression compared to the Cls group. Compared to the control group, the ethanol (ulcer) group revealed a significant drop in NO. Compared to the ulcer group, the Cls and Omp groups had a significant rise in NO contents. Conversely, the Gw pretreated Cls group showed a significant decrease in NO contents compared to the Cls group (Fig. 8C).

### Effect of Cls and Cls + Gw on gastric tissues PPAR-γ mRNA and protein expression

**Fig. 9:**
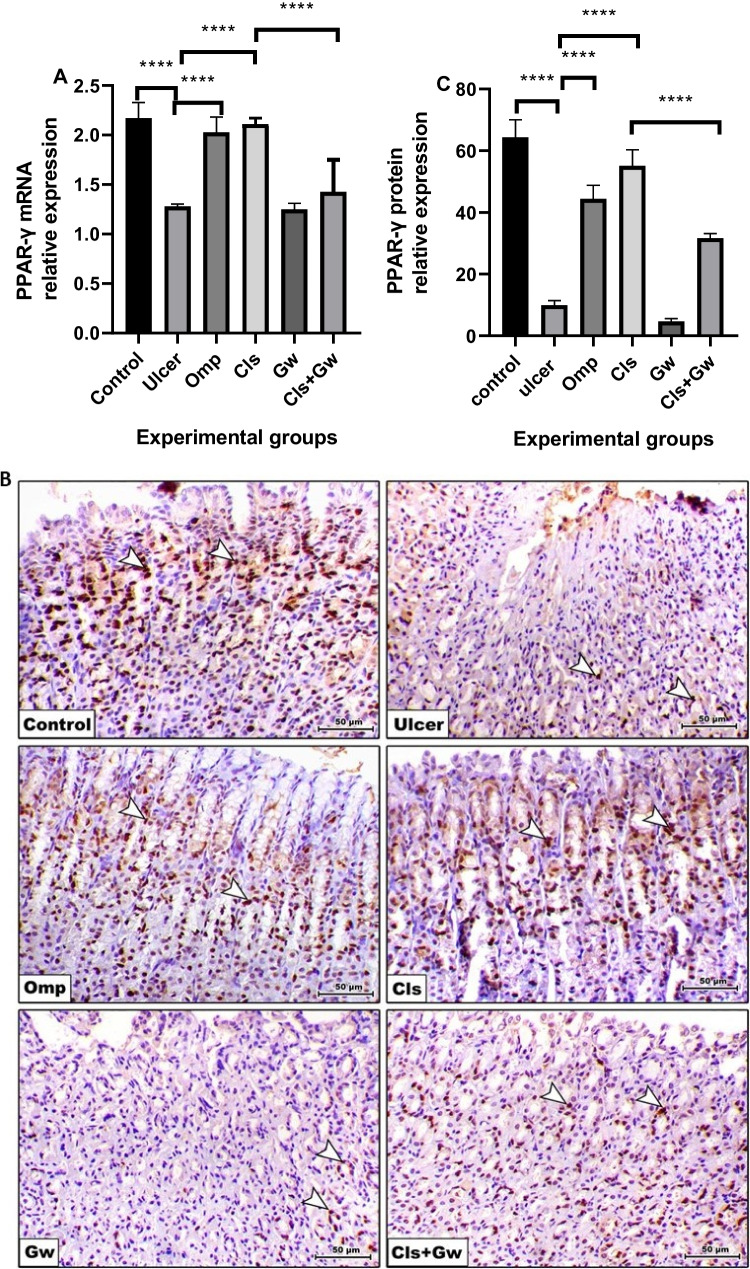
Effect of Cls and Cls + Gw on gastric tissues PPAR-γ mRNA and protein expression. **A** RT-PCR quantification of PPAR-γ mRNA; **B** immunohistochemical quantification of PPAR-γ protein (×200, bar = 50 μm); **C** Image J quantification of PPAR-γ protein. Results were presented as mean ± SD (*n* = 5 rats/group). ^****^Significance confirmed at *p* < 0.0001. Cls, cilostazol; Omp, omeprazole; Gw, GW9662

Figure 9A illustrates the gastric tissue PPAR-γ mRNA, while Fig. 9B and C illustrate PPAR-γ protein expression for each research group. Compared to the control group, the ethanol (ulcer) group revealed significant decreases in gastric PPAR-γ mRNA and protein expression. Compared to the ulcer group, the Cls and Omp groups produced significant increases in gastric PPAR-γ mRNA and protein expression. Conversely, the Gw pretreated Cls group showed a significant decrease in gastric PPAR-γ mRNA and protein expression compared to the Cls group.

### Effect of Cls and Cls + Gw on gastric tissues apoptotic cell death markers (caspase-3, cleaved caspase-3, and Bcl-2) protein expression

**Fig. 10:**
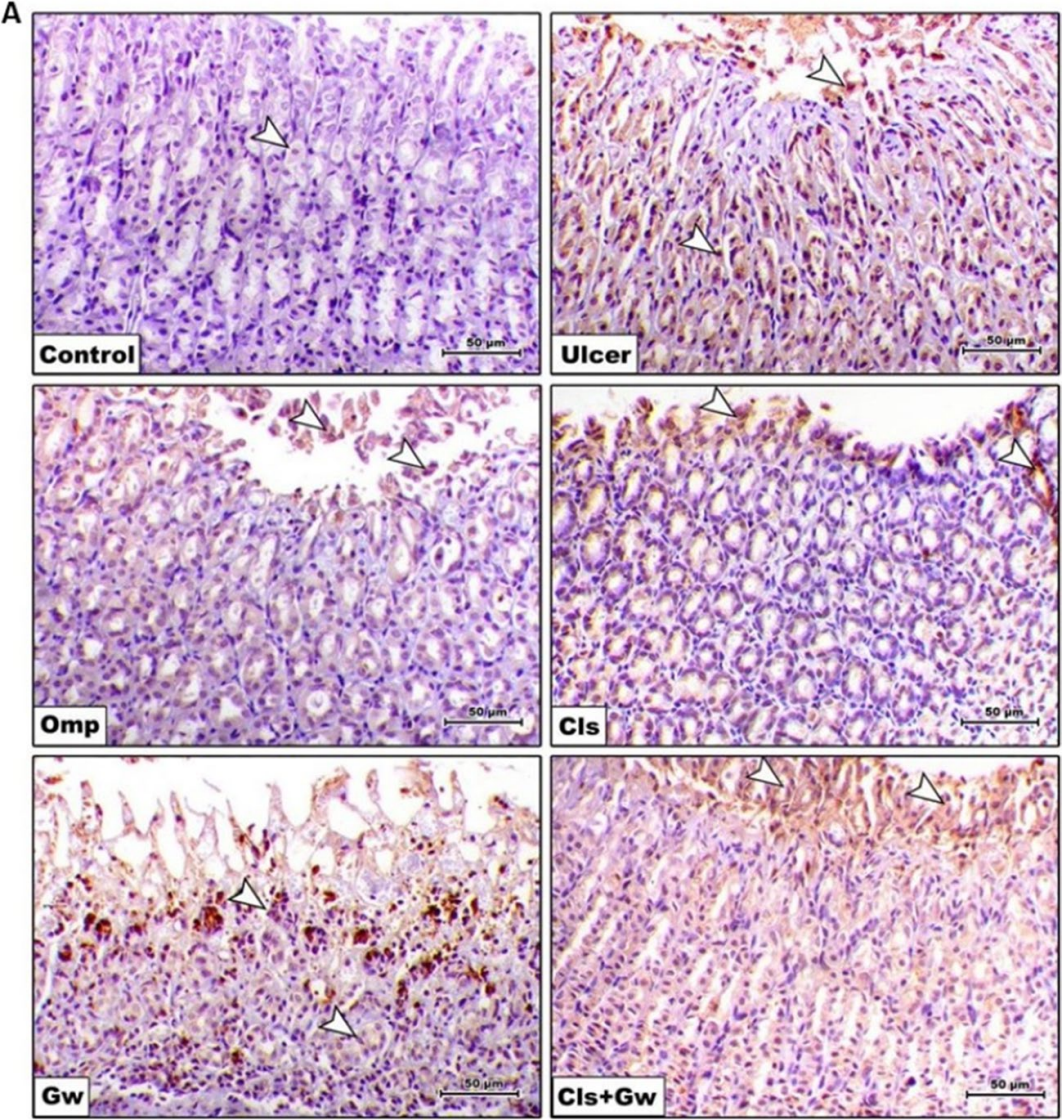

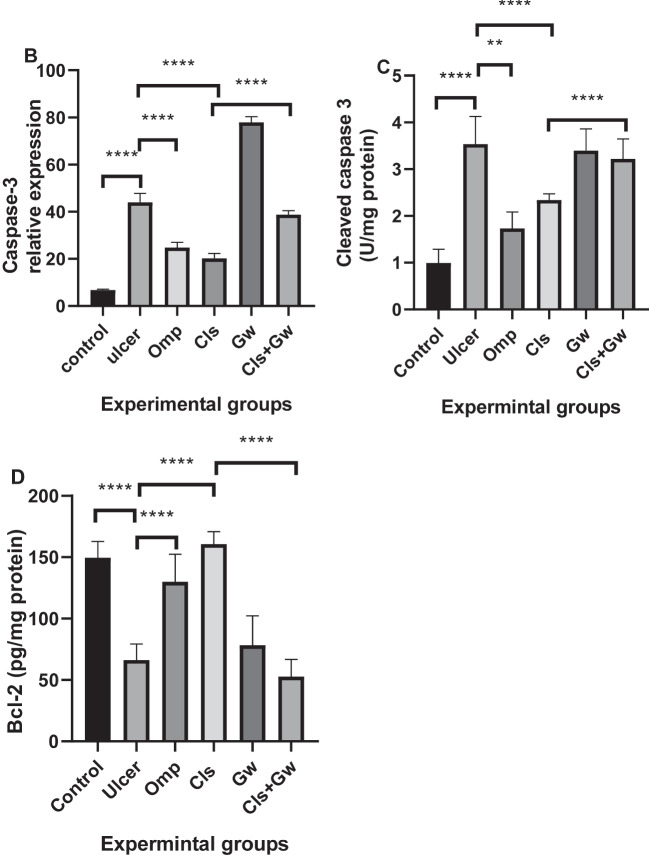
Effect of Cls and Cls + Gw on gastric tissues apoptotic cell death markers (caspase-3, cleaved caspase-3, and Bcl-2). **A** Immunohistochemical quantification of caspase-3 (×200, bar = 50 μm). **B** Relative expression of caspase-3 (Image J quantification). **C** Gastric tissue contents of cleaved caspase-3. **D** Gastric tissue contents of Bcl-2. Results were presented as mean ± SD (*n* = 5 rats/group). ^**^Significance confirmed at *p* < 0.01. ^****^Significance confirmed at *p* < 0.0001. Cls, cilostazol; Omp, omeprazole; Gw, GW9662

Figure 10A and B illustrate the gastric tissue caspase-3 immunoexpressions for each research group. Compared to the control group, the ethanol (ulcer) group revealed a significant rise in gastric caspase-3 immunoexpression. Compared to the ulcer group, the Cls and Omp groups produced significant reductions in gastric caspase-3 immunoexpression. Conversely, the Gw pretreated Cls group showed a significant increase in gastric caspase-3 immunoexpression compared to the Cls group.

Figure 10C illustrates the gastric tissue contents of cleaved caspase-3 and Fig. 10D illustrates the gastric tissue contents of Bcl-2. Compared to the control group, the ethanol (ulcer) group revealed a significant rise in gastric cleaved caspase-3 and a significant drop in gastric Bcl-2 content. Compared to the ulcer group, the Cls and Omp groups had a significant reduction in gastric cleaved caspase-3 content and a significant rise in gastric Bcl-2 content. Conversely, the Gw pretreated Cls group showed a significant increase in cleaved caspase-3 content and a significant decrease in gastric Bcl-2 content compared to the Cls group.

### Effect of Cls and Cls + Gw on gastric tissues pErk-1 protein expression

**Fig. 11:**
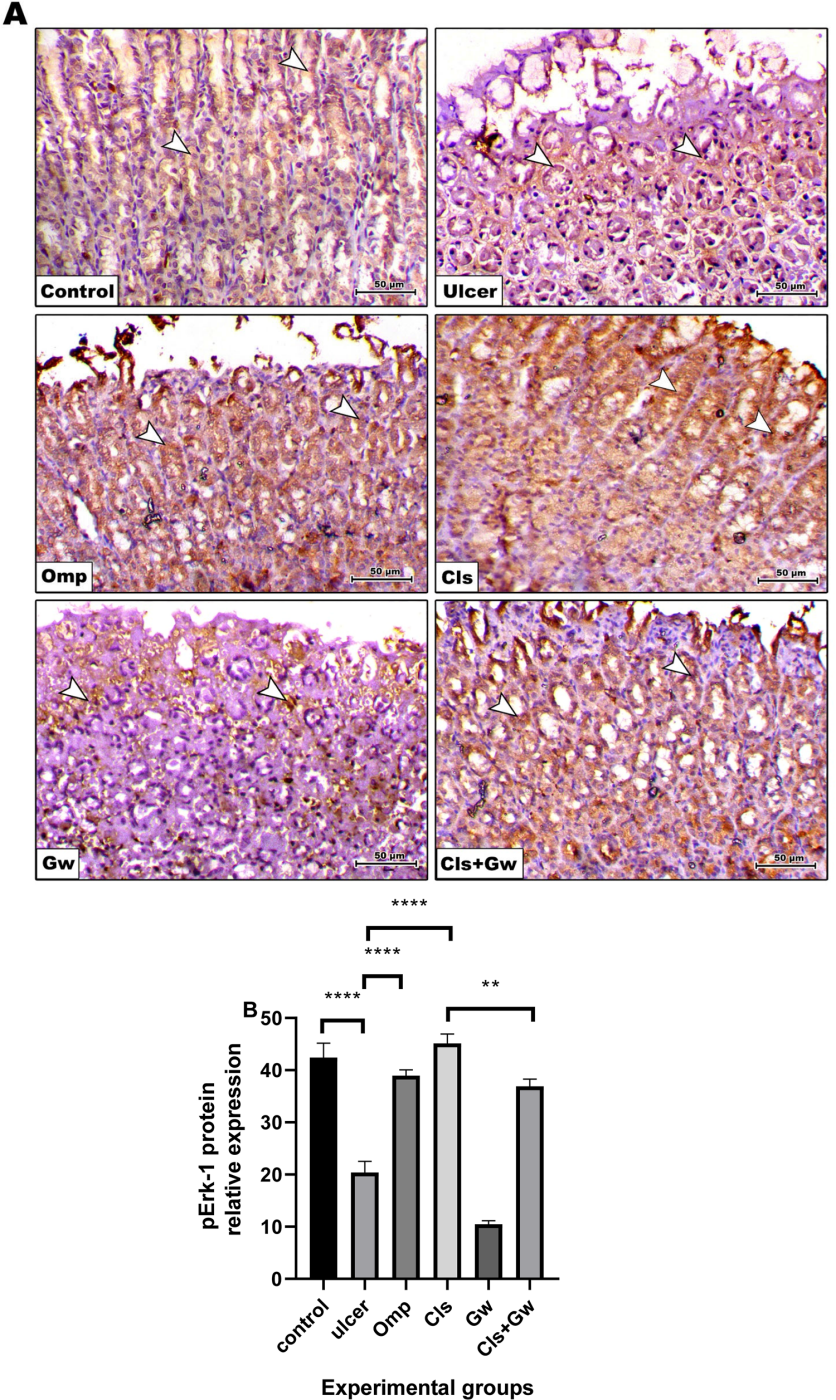
Effect of Cls and Cls + Gw on gastric tissues pErk-1 protein expression. **A** Immunohistochemical quantification of pErk-1 (×200, bar = 50 μm). **B** Relative expression of pErk-1 (Image J quantification). Results were presented as mean ± SD (*n* = 5 rats/group). ^**^Significance confirmed at *p* < 0.01. ^****^Significance confirmed at *p* < 0.0001. Cls, cilostazol; Omp, omeprazole; Gw, GW9662

Figure 11A and B illustrate the gastric tissue pErk-1 immunoexpressions for each research group. Compared to the control group, the ethanol (ulcer) group revealed a significant decrease in gastric pErk-1 immunoexpression. Compared to the ulcer group, the Cls and Omp groups produced a significant increase in gastric pErk-1 immunoexpression. Conversely, the Gw pretreated Cls group showed a significant decrease in gastric pErk-1 immunoexpression compared to the Cls group.

## Discussion

The present study demonstrated that Cls protects against ethanol-induced GU, and this effect was associated with the inhibition of oxidative stress, inflammation, and apoptosis. Importantly, the use of GW9662, a potent antagonist of PPAR-γ, reduced the antioxidant, anti-inflammatory, and antiapoptotic activities of Cls in ethanol-treated rats. The pretreatment of Cls-treated rats with PPAR-γ inhibitor significantly reversed the decrease of MDA, IL-1β, IL-6, TNF-α, NF-кB p65, and caspase-3 caused by Cls. GW9662 also decreased GSH, NO, PECAM-1, PPAR-γ, and pErk-1 levels compared to the Cls group.

Damage to the lining of the stomach is a key factor in the development of GU. This damage is caused by certain inflammatory responses that involve the accumulation of pro-inflammatory cytokines in the stomach lining as a result of ethanol consumption (Li et al. 2018). Our results showed that gavage rats with ethanol caused significant increases in the levels of the inflammatory cytokines IL-1β, TNF-α, IL-6, and NF-кB p65 expression levels in their stomach tissue. Previous studies have shown that the levels of these markers are significantly higher in the stomach tissue of rats with ethanol-induced ulcers (Su et al. 2017; Aziz et al. 2019). Treating rats with Cls before exposing them to ethanol reduced the levels of the inflammatory cytokines IL-1β, TNF-α, and IL-6 in their stomach tissue. TNF-α is a particularly important cytokine because it can cause other cytokines to be produced and can activate the NF-κB pathway, which is involved in inflammation (Sun et al. 2018; Aziz et al. 2019). Cls protects the stomach from damage caused by stress and ethanol by blocking the production of pro-inflammatory cytokines, such as TNF-α, IL-1β, and IL-6 (Ohba et al. 2006; Moawad et al. 2019). Cls protected the stomachs of both adult and old rats from the damage caused by cold restraint stress by significantly reducing the levels of TNF-α protein (Rashad et al. 2019). Cls prevent stomach ulcers caused by indomethacin by blocking the inflammatory cytokine TNF-α (Imad and Al-Qadh 2022). Cls reduces the amount of TNF-α mRNA and protein produced in murine macrophages and in the liver of mice after they are exposed to a single high dose of ethanol (Lee and Eun 2012). Cls also decreased liver content of TNF-α, IL-1β, NF-κB p65, mRNA expression levels of TNF-α and NF-κB p65, and the immunohistochemical reaction of TNF-α in thioacetamide-induced liver damage model (El Awdan et al. 2018). Blocking the enzyme type-III phosphodiesterase (PDE3) by Cls raises the levels of cAMP inside white blood cells and cells that line blood vessels. This reduces the production of the inflammatory cytokines TNF-α and IL-1β (Shin et al. 2004).

Our results showed that the gastroprotective action of Cls is associated with increased PPAR-γ mRNA and protein expression in the gastric tissue. Moreover, pretreatment of the Cls group with GW9662, a potent antagonist of PPAR-γ, significantly decreased the gastroprotective effect of Cls concomitant with the reduction in PPAR-γ mRNA and protein expression in the gastric tissue. PPAR-γ exerts anti-inflammatory and immunomodulatory effects via decreasing NF-κB p65 activity (Cuzzocrea 2004; Biscetti et al. 2013; Gendy et al. 2021) that may clarify Cls-induced modulation of TNF-α, IL-1β, and IL-6. An earlier investigation demonstrated that proangiogenic molecules like vascular endothelial growth factor (VEGF) are expressed more when PPAR-γ agonists are used (Yamakawa et al. 2000). PECAM-1 is also an important marker of gastric angiogenesis (Newman 1997). Pioglitazone, a PPAR-γ agonist, was very effective in speeding up the healing of ulcers by stimulating PECAM-1-induced angiogenesis (Brzozowski et al. 2005). Rats that were pretreated with Cls had significantly higher levels of PECAM-1 expression, which suggests that angiogenesis may play a role in the effects of Cls. This may be because Cls increases PPAR-γ activation. NO plays a vital role in angiogenesis, the repair of tissues, and the healing of ulcers (Tarnawski et al. 2014). In the present work, pretreatment with Cls caused the level of NO in the gastric tissue to increase (Moawad et al. 2019). PPAR-γ activation is associated with increased NO levels due to a decrease in NADPH oxidase activity (Nisbet et al. 2007). The current results showed that Cls downregulates caspase-3 expression and upregulates PPAR-γ expression. PPAR-γ stimulates the synthesis of Bcl-2, which impedes the release of cytochrome c and the activation caspase-3, protecting the cells from apoptosis (Wu 2010; Mahmoud-Awny et al. 2015).

Because we observed a decrease in pErk-1 expression and an increase in ROS production and caspase-3 expression in rats with ethanol-induced ulcers, we hypothesized that the decreased expression of pErk-1 may be a key mechanism underlying ethanol-induced gastric apoptosis. The pErk-1 can induce C/EBP homologous protein (CHOP) expression which may modify apoptotic cell death by preventing Akt phosphorylation. This inhibits the activity of caspases-3/9, which are enzymes that play a key role in apoptosis (Hu et al. 2019). Luo et al. (2016) showed that clopidogrel slowed down the healing of GU by inhibiting the expression of pErk over the ulcer margin and inhibiting the VEGF-VEGFR2-pErk signal transduction pathway. Moreover, increasing the expression of the pErk and Nrf2/HO-1 signaling pathways may lessen the harm produced by hypoxia-reperfusion by reducing the expression of pro-apoptotic signal factors associated with endoplasmic reticulum stress (Wang et al. 2020). Our results showed that the expression of pErk-1 and PECAM-1 were increased in rats with ethanol-induced ulcers that were pretreated with Cls. Cls also decreased the apoptotic molecule, caspase-3 immunoexpression. These suggest that Cls may promote ulcer healing by stimulating angiogenesis and inhibiting apoptosis.

## Conclusion

In this study, we proposed that the ability of Cls to block ROS, IL-1β, IL-6, TNF-α, NF-κB, and caspase-3 as well as increase pErk-1, NO, PECAM-1, and PPAR-γ levels partly explains why it protects the stomach against ethanol-induced ulcer. Cls stimulated ulcer healing and inhibited apoptosis by activating pErk-1 and PPAR-γ which led to increased PECAM-1 expression and NO level and decreased caspase-3 expression. The increase in PPAR-γ expression may be the cornerstone of Cls’s gastroprotective effect, as blocking PPAR-γ activity with GW9662 prevents Cls’s gastroprotective effect.

## Data Availability

No datasets were generated or analysed during the current study.
